# Umbilical vein marsupialization in foals—short literature review

**DOI:** 10.3389/fvets.2026.1836434

**Published:** 2026-06-30

**Authors:** Agnieszka Florczyk

**Affiliations:** Department for Small Animals and Horses, Center for Equine Health and Research, University of Veterinary Medicine Vienna, Vienna, Austria

**Keywords:** foal, marsupialization, omphalitis, omphalophlebitis, umbilical vein

## Abstract

Umbilical remnant infections are common in neonatal foals and may involve one or more umbilical structures. Diagnosis relies on thorough clinical examination, ultrasonography, and hematologic evaluation. Standard care includes broad-spectrum antibiotics, with surgical resection if conservative therapy fails. Omphalophlebitis may extend to the liver, prompting consideration of umbilical vein marsupialization. Surgery is performed in dorsal recumbency with a fusiform incision around the umbilicus. The umbilical arteries and urachus are ligated and transected, with partial cystectomy when indicated, and the umbilical vein is mobilized cranially. Marsupialization is indicated when safe ligation and transection are not feasible, or when infection extends into the hepatic parenchyma. Cranial midline translocation incorporates the partially resected vein into the cranial celiotomy closure, limiting intra-abdominal contamination. However, this approach may predispose to surgical site infection and herniation, often necessitating a second surgery. In the right paramedian translocation technique, an additional paramedian incision is created for marsupialization; the stoma heals by second intention. Although this method carries a risk of contamination during exteriorization of the vein stump; protective, risk-mitigating strategies have been reported. Perioperative management includes broad-spectrum antibiotics, ideally guided by culture and sensitivity testing, along with wound care and, in some reports, flushing of the stoma. Prognosis is less favorable for foals requiring marsupialization—typically those with hepatic involvement—than for those undergoing simple omphalectomy, although favorable long-term survival has been reported. Despite limited and heterogeneous literature, umbilical vein marsupialization remains a viable surgical option in appropriately selected cases.

## 1 Introduction

At birth, the umbilical cord in the foal divides and continues to undergo involution for 4–6 weeks (1, 2). The cord consists of the urachus, two umbilical arteries, amniotic sheath, and the umbilical vein (1, 3). Umbilical remnant infections are common in neonatal foals and can serve as portals for pathogen dissemination to other structures; consequently, effective management requiring systematic monitoring, of overall neonatal health and the umbilical stump, along with timely treatment when colostrum intake or quality is inadequate (3, 4). Clinical signs of omphalitis often include swelling and/or discharge from the external remnant. Foals may present with fever, and bloodwork often shows inflammatory changes (5). Ultrasound examination of the umbilical structures is valuable, particularly when the external stump appears normal. It allows evaluation of the progress of involution and detection of remnant infection based on heterogeneous material within the vessel lumen and/or enlargement of the structures (6, 7). The most commonly isolated bacteria are *Streptococcus equi subsp. zooepidemicus, Escherichia coli*, and *Staphylococcus aureus*. Management therefore warrants broad-spectrum antimicrobial therapy—ideally guided by culture and susceptibility testing—and surgical resection if conservative treatment fails (7–9). Published literature shows that more than one umbilical structure is often involved, with the urachus most commonly affected (7–9). Omphalophlebitis occurs less frequently than omphaloarteritis, but it can be complicated by the involvement of the liver parenchyma and abscess formation. Umbilical vein marsupialization was first described about half a century ago as alternative approach for foals and calves where resection of the affected vein was not possible (2, 10, 11). This literature review aims to compile available and yet limited information on this topic to guide clinical decision-making while comprehensive reviews regarding urogenital disorders and surgery of foals have been recently published elsewhere and are beyond the scope of this publication (5, 12).

## 2 Surgical procedure

Two approaches to umbilical vein marsupialization are described in the literature (2, 11). Both, involve positioning the animal in dorsal recumbency. A fusiform incision around the umbilicus is made, with careful dissection to avoid trauma to the umbilical remnants. Initially, the umbilical arteries and urachus are ligated, and a partial cystectomy is performed as necessary. The umbilical vein is then mobilized cranially toward the liver. Marsupialization is indicated when the extent of umbilical vein involvement precludes safe ligation and transection, or when infection already extends to the liver. Regardless of the approach, the vein is mobilized sufficiently to allow marsupialization with minimal tension.

### 2.1 Cranial midline translocation

Partial length of the vein is resected and the remaining stump incorporated into the cranial aspect of the celiotomy incision closure, frequently using two rows of interrupted sutures; however, the specific suturing technique varies across published reports. This approach avoids abdominal cavity contamination but is thought to increase the risk of surgical site infection, cellulitis, and herniation (2, 13). It requires a second surgery later to address the herniation. This technique is mostly reported in calves and less often in foals which suffered from incisional herniation, both at sites distant from and at the marsupialization site (2, 13, 14).

### 2.2 Right paramedian translocation

This technique requires an additional right paramedian incision for the umbilical vein, sparing the midline incision from the infection risk. The marsupialization site heals by second intention. Vein attachment at the stoma site varies from a single-layer (skin to the vein wall) to a two- or three-layer closure with simple interrupted sutures, incorporating the rectus sheath and subcutis with the connective tissue around the vein wall (11, 13–15). For this procedure, passing the umbilical vein stump through an incision in the body wall is necessary, which puts the abdominal cavity at risk of contamination and septic peritonitis. Various methods have been reported to protect the stump from leakage, including sutures, sterile gloves or clamps all reported as successful avoiding major contamination (11, 14).

Available literature was reviewed, and procedural variations with associated outcomes are summarized in Table 1. Given the limited evidence base and common inclusion of reports addressing foals and calves, the review encompasses publications on umbilical vein marsupialization in both species.

**Table 1:** Details of the procedure in available literature.

Publication (chronologically)	Marsupialization approach	Marsupialization technique details	Animals	Perioperative management	Outcome	Complications	Miscellaneous
Trent and Smith (10)	Right paramedian translocation	Marsupialization: 4 cm long incision 1–2 cm right paramedian and 5 cm caudal to xiphoid Stump attachment: 3 layers of SIR^a^ sutures incorporating the fibrous vein stalk to all layers of the abdominal wall	3 calves	Daily stump flushing with 10% iodine while drainage persisted. Antibiotics extended beyond 24 h postop	2 animals good or fair (drainage from the stump ceased after 12d in one calf and after 23 d in the other)	1 animal euthanized during the surgery due to excessive liver abscessation	
Steiner et al. (2)	Cranial midline translocation	Marsupialization: laparotomy incision extended to 3 cm caudal to the xiphoid, Stump attachment: vein stalk implemented in its cranial aspect with 2 rows of SIR sutures (synthetic absorbable monofilament) Other: Remainder of the celiotomy closed in 3 layers of simple continuous pattern, skin edges apposed with staples	13 calves	Daily stump flushing with 0.1% povidone (pressurized: 8 calves; non-pressurized: 5) until lavage fluid was clear for ≥3 d. Antibiotics for 3 d postop	Fair in 9 animals (8 used in breeding), poor in 2 and 2 were euthanized; based on weight gain over time in comparison to healthy calves	Stump flushing transitioned from pressurized to non-pressurized due to adverse reactions. Hernia at the marsupialization site developed in 9 animals; corrected in 8 during a second surgery; all those animals were intended for breeding	Adverse reactions to pressurized stump flushing were associated with younger age and disease severity; none occurred with non-pressurized flushing. Eight breeding-intended animals underwent corrective surgery of the marsupialization site hernia
Edwards and Fubini (11)	Right paramedian translocation	Stump attachment: 3 layers- external rectus sheath and subcutis were apposed to connective tissue of the vein wall, (synthetic absorbable monofilament 2/0 USP), skin was sutured to the wall with nonabsorbable material to be removed 14 d later, all layers closure- SIR	5 calves and 2 foals	Vein flushed with 10% povidone; venous drains placed in 2 calves; daily flushing until healed. No flushing in 1 calf and both foals due to friable veins. Antibiotics for 7–28 d	Good in all patients, some limitations of long-term survival were unrelated to the omphalophlebitis	2 calves developed cellulitis 14 d postop at marsupialization site, which resolved with repeated antibiotic therapy	Vein stump clamped as it traversed the body wall at the marsupialization site; no major breaches of sterility reported
Marchionatti et al. (14)	Right paramedian translocation	Marsupialization: incision linear/circular fitting size of the translocated vein, musle of abdominal wall disected with Metzenbaum scissors, peritoneum opened with a scalpel blade Stump attachment: one/2 layer with modified interrupted horizontal mattress (2/0–1 USP synthetic monofilament suture) incorporating the wall of the vein to the skin	20 calves (1 calf underwent cranial translocation)	In 7 calves, the stump was flushed postoperatively using a small catheter to minimize intravascular pressure Antibiotics continued ≥72 h postoperatively, extended as needed	Considered good with 74% animals performing well long term	Hernia at the marsupialization site in 6/15 calves (46%); most were small. Revision was straightforward in 4 cases; a fifth calf had a 20 cm hernia that healed by first intention without mesh. Cellulitis at the marsupialization site occurred in 13% of calves	Stump sutured and protected in a sterile glove before passage through the paramedian incision. Stump resection in the standing animal left ≤1 cm ventral to the abdominal wall, typically at 24–48 h postoperatively (range: immediate–72 h postop)
	Cranial midline translocation		1 calf			Small abdominal hernia- at the marsupialization site, not requiring surgery	
Codina et al. (8)	Cranial midline translocation	As described by Steiner et al. (see above)	2 foals		One foal developed adhesions of the operated area and jejunum, required resection and anastomosis, euthanized due to septic peritonitis		An additional foal was euthanized for severe colic; post-mortem revealed extensive hepatic infection. This foal did not undergo umbilical vein marsupialization
Klein et al. (15)	Right paramedian translocation	Marsupialization: 6 cm incision was used for marsupialization Stump attachment: 1 layer SIR, USP 2/0, synthetic monofilament material	5 foals	Stump lavage twice daily with diluted chlorhexidine during hospitalization. Antibiotics continued ≥72 h postop		3 foals developed hernia at the marsupialization site	
Obrochta et al. (13)	Right paramedian translocation	Marsupialization: 2–3 cm long right paramedian incision Stump attachment: 2 layers of SIR sutures (USP 2/0, synthetic monofilament material), vein wall to rectus sheath and vein wall to the skin	9 foals (11 foals included in the study)	Stomata cleaned superficially with 1% povidone or saline; gentle saline flush of the stump then filled with polymer hydrogel; Sutures/staples removed at 14 d Antibiotics ≥7 d postoperatively, extended as indicated Stall rest 14 d, then 6 w small paddock turnout	10/11 foals omphalophlebitis resolved and were available for long term follow up	Short-term complications: comorbidities (pneumonia/septic arthritis) in 4 foals; one euthanized 6 d after initial surgery. Other: postoperative myopathy and stoma necrosis in 1 foal. Long-term complications: hernia at the stoma site in 2 foals; hernia at the celiotomy incision in 1 foal. Colic in 1 foal	Vein ligated near the umbilicus before transection; after marsupialization, stumps were respected to within a few millimeters ventral to the abdominal wall
	Cranial midline translocation	Marsupialization: laparotomy incision extended craniad Stump attachment: vein stalk implemented in its cranial aspect with 2 rows of SIR sutures (synthetic absorbable monofilament). Other: Remainder of the celiotomy closed in 3 layers of simple continuous pattern, skin edges apposed with staples or SIR (synthetic absorbable suture)	2 foals (11 foals included in the study)			Foal 1: Hernia at caudal incision; alive at 88 m. follow-up Foal 2: Colic 4 m. postoperatively; hernia at stoma site; alive at 88 m. follow-up	

a SIR, simple interrupted suture pattern.

## 3 Perioperative management

Umbilical vein marsupialization in foals is typically undertaken after failure of conservative therapy. At presentation, bacterial infection may already have been disseminated to other sites, such as joints or lungs. Accordingly, broad-spectrum antimicrobials are required, with selection guided by bacteriologic culture and susceptibility results; however, given the severity of disease, initiation of treatment should not be delayed. Broad-spectrum antibiotics are recommended and are consistently used. Across the literature on foals with omphalitis, the most isolated organisms *are Escherichia coli, Streptococcus equi subsp. zooepidemicus, and Staphylococcus aureus* (9, 16). The duration of antibiotic therapy varies according to concurrent disease and clinical condition, and close monitoring of all affected sites is necessary. Active flushing of the marsupialized vein has been reported in both calves and foals, and it is paramount to avoid high intraluminal pressure during this procedure (2, 10, 11, 13–15). One recent publication reports superficial cleaning of the stump (13). The length of hospitalization varies according to concurrent diseases, animal’s general condition and financial constraints.

## 4 Prognosis

Foals with omphalophlebitis often suffer from other conditions, contributing to a less favorable survival rate. In addition, those requiring marsupialization- with hepatic involvement- have a less favorable prognosis than foals requiring simple omphalectomy. A study of 11 foals undergoing umbilical vein marsupialization showed a long-term survival rate of 91% (13).

## 5 Discussion

This present review highlights limited and heterogenous literature, emphasizing that umbilical vein marsupialization is much less commonly performed than simple omphalectomy. Even the most comprehensive and recent study, with evaluation period of nearly a decade and involving four referral centers, included only 11 cases (13). This small sample size in the existing literature results in difficulty drawing robust, generalizable conclusions about this procedure. Foals with severe omphalophlebitis, particularly those with extension of the infection into the liver parenchyma, exhibit a markedly more critical clinical course compared to cases managed with simple omphalectomy. More recent publications have introduced alternative techniques for handling umbilical remnants: in addition to conventional ligation, the use of vessel-sealing devices such as LigaSure has been described for transection. This approach allows resection of the umbilical vein close to the liver parenchyma; thermal damage to surrounding viscera can be minimized by protective measures such as manual shielding and silicone drape use (8, 17).

Early surgical intervention has been advocated by some authors to facilitate prompt removal of infected tissue and potentially improve outcomes. However, even with early intervention, foals presenting with significant comorbidities tend to have less favorable prognoses (7). Modifications in surgical technique, such as a two-stage approach to celiotomy wound closure with early closure of the cranial aspect following cranial vein resection or creating additional small incision at the proposed site of vein ligation have been proposed to reduce intraoperative trauma to the abdominal viscera (7).

The time lag between onset of omphalophlebitis and surgical intervention remains a consistent variability between publications. Generally, surgical treatment is indicated when conservative therapy with broad spectrum antibiotics and other supportive measures fails; nevertheless, the literature lacks definite thresholds to guide definitive decision making. Thorough evaluation of the patient suffering from omphalitis is invaluable. Furthermore, there is considerable variability of surgical details regarding attachment of the vein stump to body wall. Authors who experienced herniation at the stoma site who used single layer attachment have questioned whether multiple-layer attachment would influence this complication (14, 15). Prospective studies utilizing varied vein-to-the-body-wall attachment are lacking.

Umbilical vein marsupialization is a viable and generally safe surgical option when the liver parenchyma is involved or altered vein cannot be safely resected. Potential complications, such as hernia formation and the need for a second surgery, should be discussed with the owner, considering the cost of therapy, prognosis, and comorbid conditions.
